# Ethanol exerts dual effects on calcium homeostasis in CCK-8-stimulated mouse pancreatic acinar cells

**DOI:** 10.1186/1471-2121-10-77

**Published:** 2009-10-30

**Authors:** Marcela Fernández-Sánchez, Angel del Castillo-Vaquero, Ginés M Salido, Antonio González

**Affiliations:** 1 Department of Physiology (Cell Physiology Research Group), University of Extremadura, Cáceres, Spain

## Abstract

**Background:**

A significant percentage of patients with pancreatitis often presents a history of excessive alcohol consumption. Nevertheless, the patho-physiological effect of ethanol on pancreatitis remains poorly understood. In the present study, we have investigated the early effects of acute ethanol exposure on CCK-8-evoked Ca^2+ ^signals in mouse pancreatic acinar cells. Changes in [Ca^2+^]_i _and ROS production were analyzed employing fluorescence techniques after loading cells with fura-2 or CM-H_2_DCFDA, respectively.

**Results:**

Ethanol, in the concentration range from 1 to 50 mM, evoked an oscillatory pattern in [Ca^2+^]_i_. In addition, ethanol evoked reactive oxygen species generation (ROS) production. Stimulation of cells with 1 nM or 20 pM CCK-8, respectively led to a transient change and oscillations in [Ca^2+^]_i_. In the presence of ethanol a transformation of 20 pM CCK-8-evoked physiological oscillations into a single transient increase in [Ca^2+^]_i _in the majority of cells was observed. Whereas, in response to 1 nM CCK-8, the total Ca^2+ ^mobilization was significantly increased by ethanol pre-treatment. Preincubation of cells with 1 mM 4-MP, an inhibitor of alcohol dehydrogenase, or 10 μM of the antioxidant cinnamtannin B-1, reverted the effect of ethanol on total Ca^2+ ^mobilization evoked by 1 nM CCK-8. Cinnamtannin B-1 blocked ethanol-evoked ROS production.

**Conclusion:**

ethanol may lead, either directly or through ROS generation, to an over stimulation of pancreatic acinar cells in response to CCK-8, resulting in a higher Ca^2+ ^mobilization compared to normal conditions. The actions of ethanol on CCK-8-stimulation of cells create a situation potentially leading to Ca^2+ ^overload, which is a common pathological precursor that mediates pancreatitis.

## Background

Cholecystokinin stimulates the activity of pancreatic acinar cells via generation of different second messengers in the signal cascades [1]. The activation of phospholipase C (PLC)-linked receptors by cholecystokinin produces an increase in the concentration of inositol 1,4,5-trisphosphate (IP_3_) in the cytosol. IP_3 _in turn releases calcium (Ca^2+^) from cytoplasmic stores leading to an increase in cytosolic free calcium concentration ([Ca^2+^]_i_) [2]. In addition, a co-ordinate influx from the extracellular space [3], Ca^2+ ^extrusion across the plasma membrane [4] as well as Ca^2+ ^uptake into intracellular organelles [5] contribute to average Ca^2+ ^signals.

A rise in [Ca^2+^]_i _is an important early signal by which physiological secretagogues elicit the release of digestive enzymes from pancreatic acinar cells, being the spatiotemporal pattern of agonist-induced Ca^2+ ^signals of critical importance for exocytosis of enzymes [6]. However, although cholecystokinin is a major physiological regulator of secretion by the exocrine pancreas, an over stimulation can cause injury to the pancreas which may lead to dysfunction of the gland and even to activation of death signalling pathways involving caspases [7,8].

Additionally, an impairment of secretion would lead to intracellular activation of digestive enzymes which, in turn, could initiate auto digestion of the gland and surrounding tissues, and to the establishment of an inflammatory disease [9,10]. In relation to this, abnormally elevated [Ca^2+^]_i _has been proposed to be a shared phenomenon in acute pancreatitis that could induce trypsin premature activation [11].

It has been long recognized that a significant percentage of patients with pancreatitis often presents a history of excessive alcohol consumption. Nevertheless, the mechanisms underlying alcohol-derived deleterious effects are not completely understood.

The exocrine pancreas can metabolize ethanol mainly via an oxidative pathway involving the enzymes alcohol dehydrogenase and cytochrome P4502E1, although a nonoxidative pathway involving fatty acid ethyl ester synthases has been also proposed [12]. Therefore, metabolism of ethanol by pancreatic acinar cells and the consequent generation of toxic metabolites are postulated to play an important role in the development of alcohol-related pancreatic injury.

Reactive oxygen species (ROS) are a molecular group that can be produced in the course of different physiological processes and can react with a large variety of oxidizable cellular components. Thus, ROS have been considered as pathogenic factors in a variety of tissues and cells, pancreas inclusive [13-15]. Nowadays, there is growing evidence indicating that the exocrine pancreas is vulnerable to damage from ROS generated by ethanol metabolism [16].

One of the early events leading to alcoholic pancreatitis seems to be the effect of ethanol on stimulus-secretion coupling mechanisms. It has been suggested that ethanol acts to sensitize the pancreas to the deleterious effects of other stimuli such as the physiological agonist cholecystokinin octapeptide (CCK-8), which then leads to an inflammatory response and pancreatitis. This effect is in part mediated by augmenting activation of proinflamatory factors [17], although a decrease in the levels of prostaglandin E2 could be as well involved in alcohol-induced injury in the pancreas [18]. Furthermore, it has been proposed that ethanol induces generation of oxygen radicals in pancreatic acinar cells [19,20], induces a profound elevation of [Ca^2+^]_i _via non-oxidative metabolites [12], impairs CCK-8-evoked secretion of digestive enzymes [20] and leads to a delayed or reduced Ca^2+ ^extrusion from the cytosol towards the extracellular space or into the cytosolic stores [21]. However, it was previously proposed that ethanol alone and its oxidative metabolite acetaldehyde have minimal or no effects on Ca^2+ ^signalling [12].

We have previously hypothesized that ethanol might exert its deleterious effects in pancreatic acinar cells, at least in part, via generation of ROS, although it was not directly demonstrated [20,21]. In addition, despite the great number of investigations carried out to study the action of ethanol on cell physiology, the involvement of Ca^2+ ^metabolism in ethanol-evoked effects needs to be further studied, because most cellular activity is initiated by changes in [Ca^2+^]_i_. In the present study, we have investigated the early effects of acute ethanol exposure on CCK-8-evoked Ca^2+ ^signals in mouse pancreatic acinar cells. Our objective was to shed more light onto the effects of ethanol on the physiological action of CCK-8. The findings will contribute to a better understanding of the mechanisms by which ethanol causes pancreatic disorders.

## Results

### Changes in [Ca^2+^]_i _in response to CCK-8 and effects of ethanol in single cell studies

**Figure 1:**
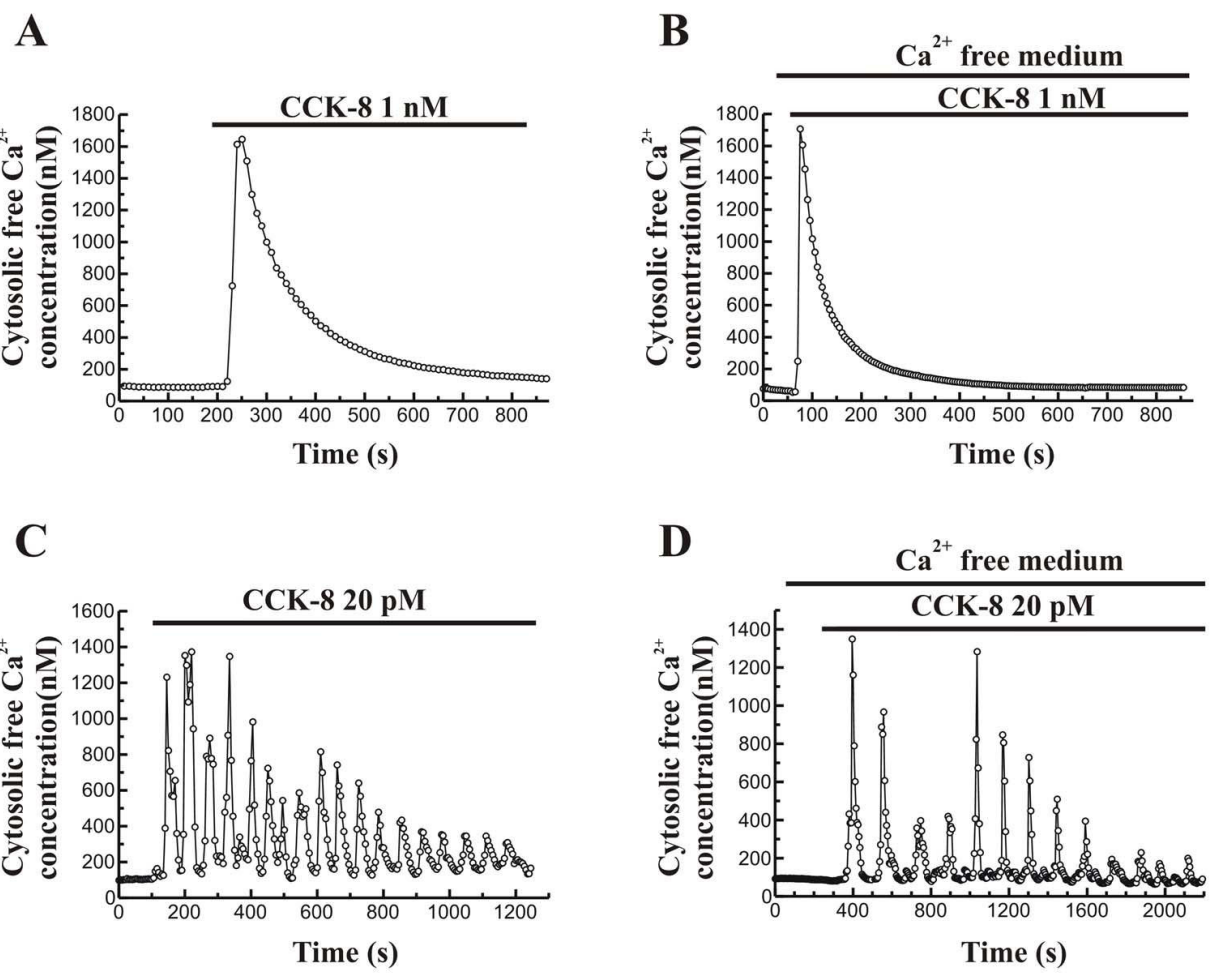
**Time-course of changes in [Ca^2+^]_i _in response to CCK-8**. Fura-2 loaded pancreatic acinar cells were stimulated with 1 nM CCK-8 in the presence (A) and in the absence (B) of Ca^2+ ^in the extracellular medium. On the other hand, cells were stimulated with 20 pM CCK-8 in the presence (C) and in the absence (D) of Ca^2+ ^in the perfusion medium. The horizontal bars indicate the time during which CCK-8 was applied to the cells. The traces are typical of 3-5 independent experiments.

As it has been previously shown [21], stimulation of fura-2-loaded cells with 1 nM CCK-8, in the presence of extracellular Ca^2+^, led to a transient increase in [Ca^2+^]_i_. Ca^2+ ^mobilization consisted of an initial increase followed by a decrease of [Ca^2+^]_i _towards a value close to the prestimulation level (n = 3 experiments/26 cells studied; Figure 1A). This pattern of Ca^2+ ^mobilization was also observed in the absence of Ca^2+ ^in the extracellular medium, although, in these conditions, the recovery phase was faster (n = 3 experiments/23 cells studied; Figure 1B).

When pancreatic acinar cells were stimulated with 20 pM CCK-8, Ca^2+ ^mobilization depicted an oscillatory pattern, which was observed either in the presence (n = 5 experiments/24 cells studied; figure 1C) and in the absence (n = 3 experiments; 25 cells studied; figure 1D) of extracellular Ca^2+^. These patterns of changes in [Ca^2+^]_i _have been previously observed [1,5].

**Figure 2:**
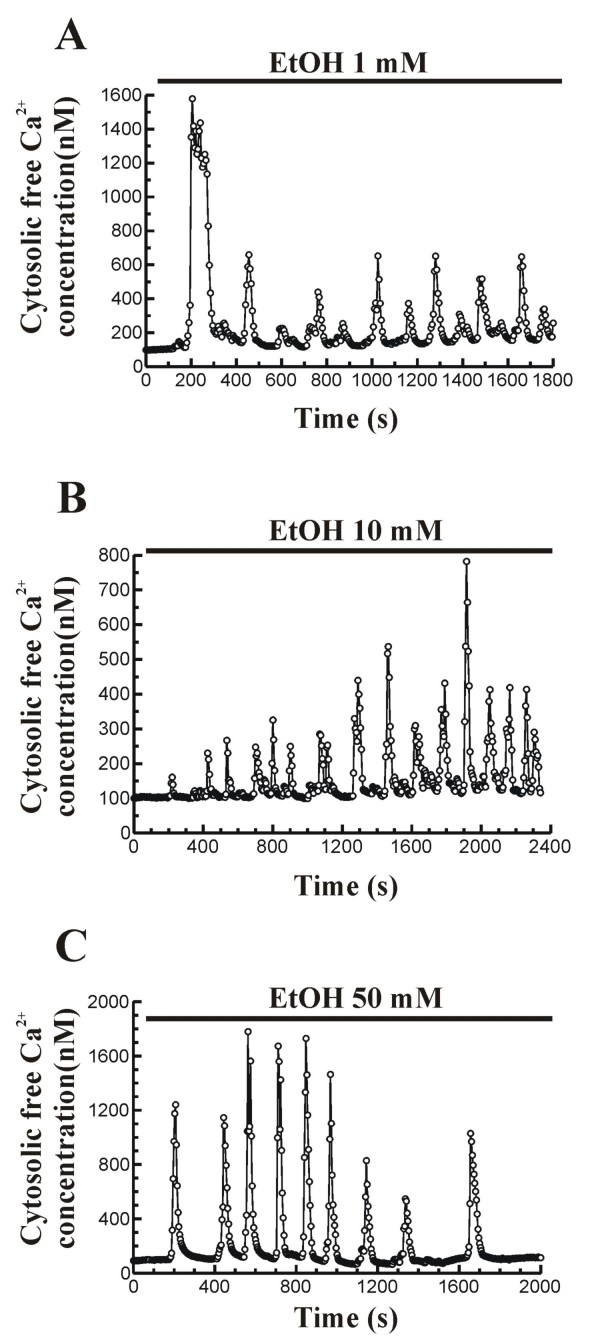
**Time-course of changes in [Ca^2+^]_i _in response to ethanol**. Fura-2 loaded pancreatic acinar cells were perfused with 1 mM (A), 10 mM (B) or 50 mM ethanol (EtOH) in the presence of Ca^2+ ^in the extracellular medium. The horizontal bars indicate the time during which ethanol was applied to the cells. The traces are typical of 4-5 independent experiments.

Perfusion of pancreatic acinar cells with ethanol in the presence of Ca^2+ ^in the extracellular medium, led to Ca^2+ ^mobilization in the form of oscillations (Figure 2). These changes in [Ca^2+^]_i _were evoked by 1 mM (n = 6 experiments/49 cells studied), 10 mM (n = 4 experiments/27 cells studied) and 50 mM ethanol (n = 5 experiments/24 cells studied), and were also observed in the absence of external Ca^2+ ^(data not shown). The effects of ethanol on [Ca^2+^]_i _did not depict a concentration-dependent effect. In addition, it was not possible to define a pattern for Ca^2+ ^mobilization induced by ethanol, because it varied in terms of frequency and amplitude of oscillations, independently of the concentration employed and even at the same concentration of ethanol.

Throughout the following determinations, we chose a 50 mM ethanol concentration, since it is a concentration falling within the range of blood alcohol levels in intoxicated humans [22]. In addition, this concentration of ethanol clearly affected Ca^2+ ^transport mechanisms and amylase secretion in response to CCK-8 in pancreatic acinar cells [20,21].

**Figure 3:**
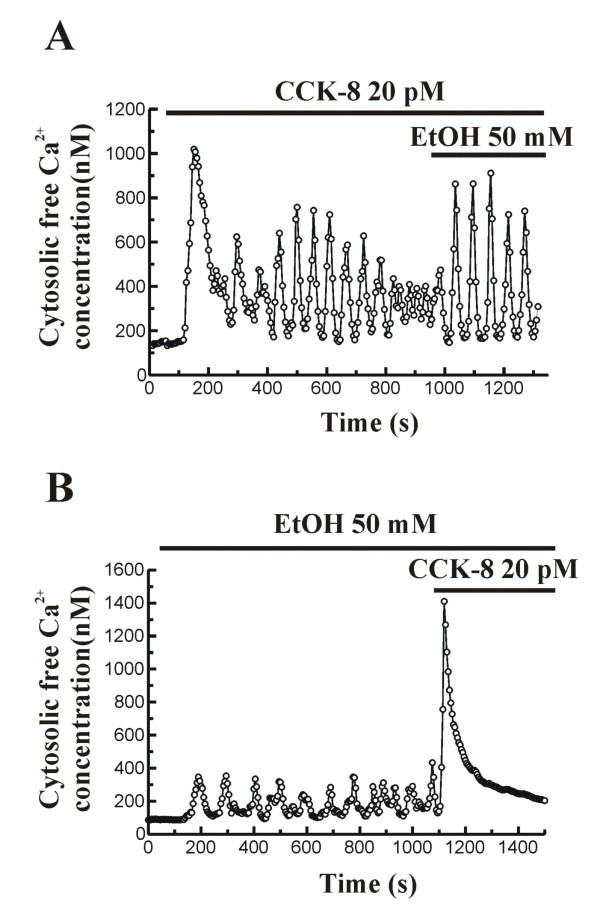
**Time-course of CCK-8-evoked changes in [Ca^2+^]_i _in the presence of ethanol**. (A) Fura-2 loaded pancreatic acinar cells were perfused with 20 pM CCK-8 in the presence of Ca^2+ ^in the extracellular medium. Following CCK-8 stimulation, 50 mM ethanol (EtOH) was included in the perfusion medium. (B) Pancreatic acinar cells were perfused with 50 mM ethanol (EtOH) in the presence of Ca^2+ ^in the extracellular medium. In the presence of ethanol 20 pM CCK-8 was included in the perfusion medium. The horizontal bars indicate the time during which ethanol and CCK-8 were applied to the cells. The traces are typical of 8-9 independent experiments.

In another set of experiments, we evaluated the effect of ethanol on CCK-8-evoked Ca^2+ ^signals. In the presence of extracellular Ca^2+^, perfusion of cells with 20 pM CCK-8 induced oscillations in [Ca^2+^]_i _as we have shown above. The amplitude of oscillations progressively decreased after 15 minutes in the presence of the peptide. Under these conditions, inclusion of 50 mM ethanol in the perfusion medium induced a potentiation of CCK-8-evoked oscillations in [Ca^2+^]_i_, observed as an increase in the amplitude of Ca^2+ ^spikes (n = 8 experiments/83 cells of 124 total cells studied; Figure 3A).

On the other hand, when 50 mM ethanol was first applied to the cells, the oscillatory mobilization of [Ca^2+^]_i _that we have shown was observed. In the presence of ethanol (50 mM), perfusion of cells with 20 pM CCK-8 evoked an oscillatory patter of [Ca^2+^]_i _over an elevated level in the 18.27% of cells (n = 9 experiments/21 cells of 115 total cells studied). The majority of cells (81.73%) showed a single transient change in [Ca^2+^]_i_, similar to that evoked by the supramaximal concentration of 1 nM CCK-8 (n = 9 experiments/94 cells of 115 total cells studied; Figure 3B).

### Changes in [Ca^2+^]_i _in response to CCK-8 and effect of ethanol in cell-suspension studies

To better analyze the effects of ethanol on CCK-8 evoked Ca^2+ ^mobilization, we performed a series of experiments on cell suspensions, where the behaviour of a population of 10^6 ^cells/ml was studied. Throughout these experiments, pancreatic acinar cells were stimulated with 1 nM CCK-8 alone or in the presence of 50 mM ethanol.

**Figure 4:**
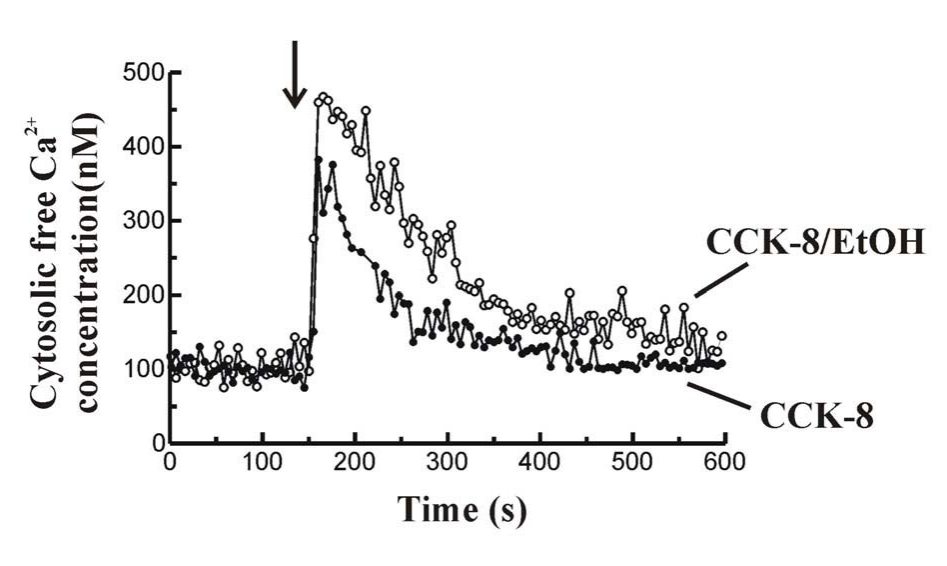
**Changes in [Ca^2+^]_i _in response to CCK-8 and effect of ethanol**. Time-course of changes in [Ca^2+^]_i _in fura-2-loaded mouse pancreatic acinar cells. Cells were stimulated with 1 nM CCK-8 alone (full circles) or in the presence of 50 mM ethanol (EtOH; open circles) in the presence of Ca^2+ ^in the extracellular medium. The arrow indicates the time point at which CCK-8 was added to the cells. In the experiments in which its effect was assayed, EtOH was added to the cells 1 minute prior to addition of CCK-8. The traces are typical of 14-17 such independent experiments.

The time course of changes in [Ca^2+^]_i _following stimulation of cells with 1 nM CCK-8 in the presence of 50 mM ethanol can be seen in figure 4.

Stimulation of cells with 1 nM CCK-8 in the presence of extracellular Ca^2+^, led to the typical initial increase followed by a decrease of [Ca^2+^]_i _towards a value close to the prestimulation level. The maximal [Ca^2+^]_i _calculated at the peak obtained after stimulation of cells with CCK-8 was 314.30 ± 17.39 nM (n = 17). [Ca^2+^]_i _reached a steady-state value of 136.30 ± 7.75 nM (n = 17), calculated 5 minutes after stimulation with the peptide. The total Ca^2+ ^mobilization after the stimulation was estimated to be 27930 ± 2801 nM (n = 17), whereas the decay constant of [Ca^2+^]_i _to the basal value was 0.0093 ± 0.0014 Δnmol/s (n = 17).

When pancreatic acinar cells were stimulated with CCK-8 (1 nM) after 1 minute preincubation in the presence of 50 mM ethanol, the peak of [Ca^2+^]_i _observed was higher compared to that obtained with CCK-8 alone (462.10 ± 22.26 nM, n = 14 vs 314.30 ± 17.39 nM, n = 17; *P *< 0.001). In the presence of ethanol, [Ca^2+^]_i _reached a significantly higher steady-state value compared to that found in the presence of CCK-8 alone (173.10 ± 9.76 nM, n = 14 vs 136.30 ± 7.75 nM, n = 17; *P *< 0.01).

The total Ca^2+ ^mobilization evoked by CCK-8 in the presence of ethanol was significantly increased compared to CCK-8 alone (43380 ± 4844 nM, n = 14 vs 27930 ± 2801 nM, n = 17; *P *< 0.01). In addition, the decay constant of [Ca^2+^]_i _to the basal value was slowed down in the presence of ethanol, compared to decay observed after application of CCK-8 alone (0.0051 ± 0.0004 Δnmol/s, n = 14 vs 0.0093 ± 0.0014 Δnmol/s, n = 17; *P *< 0.05).

### Effect of alcohol dehydrogenase inhibition on ethanol-evoked changes in [Ca^2+^]_i _in response to CCK-8

Alcohol dehydrogenase (ADH) plays an important role in the metabolism of alcohol. The following set of experiments was carried out in order to evaluate whether the effects of ethanol are direct or not. In order to characterize the differences in Ca^2+ ^responses between the control experiments with CCK-8 alone and those performed in the presence of ethanol, it was of interest to investigate the changes in [Ca^2+^]_i _after inhibition of ADH. These experiments were performed on cell suspensions.

Under these experimental conditions, cells were preincubated for 30 minutes in the presence of the ADH inhibitor 4-methylpyrazole (4-MP) [23-25].

When cells were challenged with 1 nM CCK-8 in the presence of 1 mM 4-MP and following 1 minute preincubation with 50 mM ethanol, the peak [Ca^2+^]_i _response and the total Ca^2+ ^mobilization were significantly increased compared to the values obtained in the presence of CCK-8 alone. However, the "steady-state level" and the rate of decay of [Ca^2+^]_i _to basal values did not differ statistically from the values obtained in the presence of CCK-8 alone (Table 1).

**Table 1:** Changes in [Ca^2+^]_i _in response to CCK-8 alone and in the presence of ethanol, and effect of alcohol dehydrogenase inhibition by 4-MP and cinnamtannin B-1

	**CCK-8**	**CCK-8/EtOH**	**CCK-8/EtOH/4-MP**	**CCK-8/EtOH/CinB**
Peak [Ca^2+^]_i _(nM)	314.30 ± 17.39 n = 17	462.10 ± 22.26 n = 14 ***	469.40 ± 15.30 n = 7 ***	448.00 ± 19.99 n = 11 *

Steady level (nM)	136.30 ± 7.75 n = 17	173.10 ± 9.76 n = 14 **	140.10 ± 9.64 n = 7 †	134.40 ± 11.21 n = 11 ††

Rate of decay (Δnmol/s)	0.0093 ± 0.0014 n = 17	0.0051 ± 0.0004 n = 14 *	0.0105 ± 0.0014 n = 7 ††	0.0077 ± 0.0006 n = 11 ††

Total Ca^2+ ^(nM)	27930 ± 2801 n = 17	43380 ± 4844 n = 14 **	37470 ± 2010 n = 7 *	39610 ± 5163 n = 11 *

Pancreatic acinar cells were stimulated with 1 nM CCK-8 alone, in the presence of 50 mM ethanol (EtOH), after preincubation in the presence of 1 mM of the alcohol dehydrogenase inhibitor 4-MP or following preincubation in the presence of 10 μM of the antioxidant cinnamtannin B-1 (CinB). Following CCK-8 stimulation, the peak [Ca^2+^]_i _response, the "steady-state level" achieved five minutes after application of the stimulus, the total Ca^2+ ^mobilization and the rate of decay of [Ca^2+^]_i _towards basal values were calculated (*, P < 0.05; **, P < 0.01; ***, P < 0.001 vs CCK-8; †, P < 0.05; ††, P < 0.01 vs CCK-8 plus EtOH).

On the other hand, inhibition of ADH with 1 mM 4-MP in the presence of 50 mM ethanol, did not significantly change the CCK-8-evoked peak [Ca^2+^]_i _response compared to that obtained after stimulation of pancreatic acinar cells with the hormone in the presence of 50 mM ethanol. However, the "steady-state level" was significantly reduced and the rate of decay of [Ca^2+^]_i _to basal values was significantly increased, which resulted in a smaller total Ca^2+ ^mobilization compared to the responses evoked by 1 nM CCK-8 in the presence of 50 mM ethanol alone (Table 1).

### Effect of ethanol on ROS production

**Figure 5:**
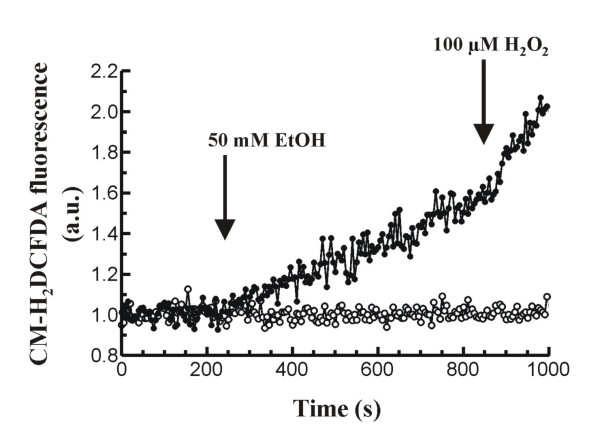
**Time-course of ROS production in mouse pancreatic acinar cells**. Stimulation of cells with 50 mM ethanol (EtOH) led to a significant increase in ROS generation over the basal (resting) value (full circles). In the presence of the antioxidant cinnamtannin B-1 (10 μM) alcohol-evoked response was significantly decreased (open circles). Stimuli were added to the cuvette at the time points indicated by arrows. At the end of the experiment 100 μM H_2_O_2 _was added to the cells as a control to show increased fluorescence following oxidation of the probe by H_2_O_2_. Graphics are representative of 6-8 such experiments.

It has been shown that ethanol leads to ROS production in mouse pancreatic acinar cells [20]. To confirm this previous observation, we stimulated mouse pancreatic acinar cells loaded with the ROS-sensitive fluorescent probe CM-H_2_DCFDA with 50 mM ethanol in the presence of extracellular Ca^2+^. Under these conditions, ethanol led to a statistically significant increase in fluorescence, suggesting oxidation of the dye in response to ethanol. The time-course of changes in fluorescence of CM-H_2_DCFDA-loaded cells in response to ethanol can be seen in figure 5. After 10 minutes in the presence of ethanol a value of 1.433 ± 0.097 a.u. (n = 8) was achieved, which was significantly higher compared to the basal value (0.998 ± 0.002 a.u., n = 6; *P *< 0.001). At the end of the experiment, 100 μM H_2_O_2 _was added to the cells as a control to show increased fluorescence following additional oxidation of the probe by exogenous H_2_O_2_.

In another set of experiments, cells were preincubated in the presence of 10 μM of the antioxidant cinnamtannin B-1. This substance is a naturally occurring A-type proanthocyanidin, which belongs to a class of polyphenols that is widely distributed throughout the plant kingdom. These compounds have long been investigated due to the antioxidant functions, which have been shown to involve radical scavenging, quenching, and enzyme-inhibiting actions [26].

Pretreatment with 10 μM cinnamtannin B-1 significantly reduced ethanol-evoked increase in CM-H_2_DCFDA-derived fluorescence by a 89.43%, although it failed to completely abolish ethanol response on ROS production (1.044 ± 0.016 a.u., n = 6 vs 1.433 ± 0.097 a.u., n = 8; P < 0.01; Figure 5).

### Effect of ethanol-induced ROS generation on CCK-8-evoked changes in [Ca^2+^]_i_

**Figure 6:**
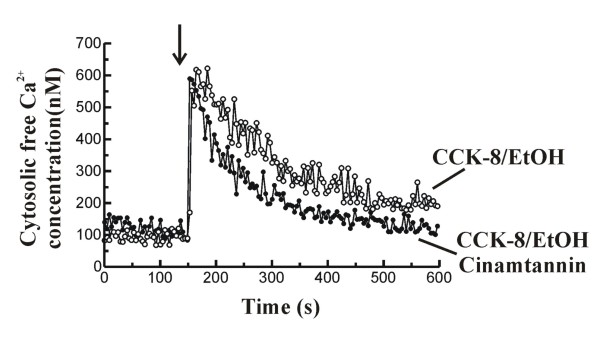
**Changes in [Ca^2+^]_i _in response to CCK-8 and effect of ethanol and cinnamtannin B-1**. Time-course of changes in [Ca^2+^]_i _in fura-2-loaded mouse pancreatic acinar cells. Cells were stimulated with 1 nM CCK-8 in the presence of 50 mM ethanol (EtOH), added 1 minute prior to CCK-8, and after 30 minutes preincubation in the presence of 10 μM cinnamtannin B-1 (full circles), or only in the presence of 50 mM ethanol (open circles). Experiments were carried out in the presence of Ca^2+ ^in the extracellular medium. The arrow indicates the time point at which CCK-8 was added to the cells. The traces are typical of 11-14 such independent experiments.

In order to evaluate whether the effects of ethanol on [Ca^2+^]_i _are mediated by a secondary ROS generation, we investigated the changes in [Ca^2+^]_i _in the presence of the antioxidant cinnamtannin B-1. The time course of changes in [Ca^2+^]_i _following stimulation of cells with 1 nM CCK-8 in the presence of 50 mM ethanol and in the presence of 10 μM cinnamtannin B-1 can be seen in figure 6.

When cells were challenged with 1 nM CCK-8 after preincubation during 30 minutes in the presence of 10 μM cinnamtannin B-1, and following 1 minute preincubation with 50 mM ethanol, the peak [Ca^2+^]_i _response and the total Ca^2+ ^mobilization were significantly increased compared to the values obtained in the presence of CCK-8 alone. However, the "steady-state level" and the rate of decay of [Ca^2+^]_i _to basal values did not differ statistically from the values obtained in the presence of CCK-8 alone (Table 1).

On the other hand, stimulation of cells with 1 nM CCK-8 in the presence of 50 mM ethanol, and after 30 minutes preincubation in the presence of cinnamtannin B-1 (10 μM), did not significantly change the CCK-8-evoked peak [Ca^2+^]_i _response compared to that obtained after stimulation of pancreatic acinar cells with the hormone in the presence of 50 mM ethanol alone. However, the "steady-state level" was significantly decreased and the rate of decay of [Ca^2+^]_i _to basal values was significantly increased in the presence of cinnamtannin B-1, which resulted in a smaller total Ca^2+ ^mobilization compared to the responses evoked by 1 nM CCK-8 in the presence of 50 mM ethanol alone (Table 1).

## Discussion

Alcohol consumption has long been associated with cell damage. Many studies on ethanol toxicity have been conducted following middle or long term exposition to ethanol [18,27-30]. However, attention on the early steps initiated by ethanol is currently lacking. Furthermore, although relevant studies have been performed on the effect of ethanol on the oxidative state of cellular components and/or apoptosis [7,8,16,28], its effects on Ca^2+ ^homeostasis have not received much attention.

To date, it is not clear whether the deleterious actions of ethanol on cellular physiology could result from the production of toxic derivatives from alcohol metabolism or from a direct action of alcohol on cellular structures, which would be morphologically and functionally altered [12,16,27,30-33].

Recently we have shown that ethanol, leading to a delayed or reduced Ca^2+ ^extrusion from the cytosol towards the extracellular space or into the cytosolic stores, induces a cytosolic Ca^2+ ^overload that could be the basis of alcoholic pancreatitis [21]. However, the mechanisms by which ethanol exerted its effect was not clearly demonstrated, although it was postulated that ROS generation might be involved in the process.

In the present study, we have further investigated the possible mechanisms involved in the early effects of an acute ethanol exposure on CCK-8-evoked Ca^2+ ^signals in mouse pancreatic acinar cells, because Ca^2+ ^signalling is of critical importance for CCK-8-evoked responses in the exocrine pancreas.

Our results show that ethanol induces mobilization of Ca^2+ ^in the form of oscillations, a pattern of Ca^2+ ^mobilization that seems not to depend on the concentration of ethanol applied to the cells. To our knowledge, this is the first time to be shown that ethanol induces Ca^2+ ^mobilization in pancreatic acinar cells at such concentrations, 1 to 50 mM, being the highest a concentration of ethanol found in plasma of intoxicated humans [22]. In a former work, higher concentrations of ethanol were employed, up to 850 mM, and effects of lower concentrations were discarded [12]. In addition, here we show that ethanol leads to a transformation of the typical oscillatory pattern of [Ca^2+^]_i_, evoked by a physiological concentration of CCK-8, into a sustained response usually evoked by supraphysiological concentrations of the agonist. Our results support previously reported works, which propose that ethanol-induced sensitization of the pancreatic acinar cell results in pancreatitis responses with low doses of CCK-8, that by itself does not cause pancreatitis [17]. Our observations further support those shown by Petersen and Sutton [31], who propose that cell death results from excessive loss of Ca^2+ ^from the endoplasmic reticulum, which is mediated by Ca^2+ ^release through specific channels and inhibition of Ca^2+ ^pumps in intracellular stores, followed by entry of extracellular Ca^2+^. All this together leads to abnormal global and sustained cytosolic Ca^2+ ^signals.

In the present work, we have further studied the mechanisms by which ethanol evokes its deleterious effects in pancreatic acinar cells, in order to clarify whether the effects of ethanol are direct or mediated through its metabolization, and if ROS are somehow involved.

The inhibition of alcohol metabolization would increase those effects that are due to a direct action of ethanol, whereas it would block those effects of ethanol that would be due to its metabolites. Through our study, we have employed the ADH inhibitor 4-MP. This compound substantially decreased ethanol effects on connective tissue growth factor mRNA expression in mouse pancreatic stellate cells [25]. Inhibition of ADH by 4-MP is concentration-dependent, with a range of action from 1 μM up to 1000 μM in different tissues [23,24,34]. Thus, the concentration of the ADH inhibitor we have employed falls within the concentration range successfully employed to inhibit ethanol metabolization by this enzyme.

Our results show that the effect of ethanol on the peak [Ca^2+^]_i _response evoked by CCK-8 was not blocked by the inhibition of ethanol metabolization by ADH; conversely, it was slightly increased. However, the "steady-state level" and the rate of decay of [Ca^2+^]_i _to basal values after CCK-8 stimulation in the presence of ethanol were significantly changed, i.e., [Ca^2+^]_i _recovered faster in the presence of ADH inhibition.

Our results could be explained on the basis of a direct action of ethanol onto the Ca^2+^-releasing mechanisms activated by CCK-8, and by an indirect action, mediated through ethanol metabolites, on the [Ca^2+^]_i _recovery mechanisms. We think that ethanol itself sensitizes the pancreas to the effects of other stimuli such as the physiological agonist CCK-8, acting on CCK-8-receptor or on the Ca^2+ ^releasing channels. This could initially have an effect on the Ca^2+ ^releasing mechanisms, and therefore would result in a bigger Ca^2+ ^response of the tissue. This is reflected as a transformation of the physiological oscillations in [Ca^2+^]_i _into a supraphysiological sustained change in [Ca^2+^]_i_, and by the higher peak of [Ca^2+^]_i _observed in the presence of ethanol.

On the other hand, the recovery of [Ca^2+^]_i _is mainly carried out by the sarco-endoplasmic reticulum Ca^2+^-ATPase and the plasma membrane Ca^2+^-ATPase, the pumps that actively transport Ca^2+ ^into the endoplasmic reticulum and towards the extracellular medium respectively. A delay or decreased extrusion of Ca^2+ ^from the cytosol will lead to an increased mobilization of Ca^2+ ^compared to normal conditions. This can potentially lead to a cytosolic Ca^2+ ^overload. Thus, t decrease in the generation of ethanol metabolites by inhibition of its metabolization would allow the Ca^2+ ^transport mechanisms to effectively recover resting [Ca^2+^]_i_, as we have shown in the present study.

This is in agreement with our previous findings, which show that ethanol slows down Ca^2+ ^transport into the ER and through the plasma membrane, which potentially leads to a cytosolic Ca^2+ ^overload [21]. However, the exact mechanisms by which ethanol evokes these effects need to be further clarified.

The antioxidant activity of proanthocyanidins is stronger than vitamin C or vitamin E in aqueous systems and its protective effects on diseases related to ROS have been demonstrated in a number of cell types [35-37]. Our results show that ethanol induces ROS production in mouse pancreatic acinar cells, in agreement with previous studies [20]. Moreover, preincubation of pancreatic acinar cells in the presence of cinnamtanninB-1 significantly inhibited ethanol-stimulated ROS generation. This supports the hypothesis that ethanol metabolization could lead to ROS generation which, in turn, would affect Ca^2+ ^transport mechanisms, and is in agreement with our previous findings [20,21].

On the other side, preincubation of pancreatic acinar cells in the presence of this antioxidant did not block ethanol-induced increase in the peak of [Ca^2+^]_i _in response to CCK-8. This further supports the hypothesis that ethanol might be having direct effects on the Ca^2+^-releasing mechanisms in response to CCK-8, as suggested above. However, in the presence of the antioxidant, the "steady-state level" achieved after stimulation of cells with the hormone was lowered compared to that observed in the presence of ethanol, and the rate of decay of [Ca^2+^]_i _to basal values after CCK-8 stimulation was faster than in the presence of ethanol, reaching values similar to those observed after stimulation of cells with CCK-8 alone. This supports the hypothesis that ethanol metabolization could lead to ROS generation which, in turn, would affect Ca^2+ ^transport mechanisms.

Therefore, here we can hypothesize that ethanol influences CCK-8-evoked changes in [Ca^2+^]_i _both by a direct and an indirect action, the latter mediated through ROS generation. Our findings are in agreement with others which show that ethanol can induce several cellular reactions which result in a modification of cellular red-ox status, leading to overproduction of ROS [16,19,38]. Nevertheless, involvement of non-oxidative metabolites from ethanol cannot be excluded.

In summary, our findings show that ethanol has dual effects on the physiology of intracellular Ca^2+ ^signalling. First, ethanol induces a direct effect on the Ca^2+ ^release mechanisms in response to CCK-8; and second, ethanol presents an indirect effect that is mediated, at least in part, by ROS generation following its metabolization. All this together leads to higher levels of [Ca^2+^]_i _following stimulation of cells with CCK-8. Ethanol will consequently lead to Ca^2+ ^accumulation within the cytosol, creating a situation potentially leading to cytosolic Ca^2+ ^overload, which is a common pathological precursor that mediates pancreatitis.

## Conclusion

Ethanol may lead, either directly or through ROS generation, to an over stimulation of pancreatic acinar cells in response to CCK-8, resulting in a higher Ca^2+ ^mobilization compared to normal conditions. The actions of ethanol on CCK-8-stimulation of cells create a situation potentially leading to Ca^2+ ^overload, which is a common pathological precursor that mediates pancreatitis.

## Methods

### Animals and chemicals

Adult male *Swiss *mice were used for this study. Mice were humanely handled and sacrificed in accordance to the institutional Bioethical Committee. Fura-2/AM was obtained from Invitrogen (Spain) and cinnamtannin B-1 from Alexis Corporation (Switzerland). All other materials used were obtained from Sigma Chemicals Co. (Spain).

### Preparation of isolated pancreatic acinar cells

A suspension of mouse pancreatic single cells and small acini (2-4 cell clusters) was prepared by collagenase treatment following a previously described method [5]. Briefly, the pancreas was incubated in the presence of collagenase for 10 min at 37°C. This enzymatic digestion of the tissue was followed by gently pipetting the cell suspension through tips of decreasing diameter for mechanical dissociation of the cells. After centrifugation, cells were resuspended in a buffer without collagenase. With this isolation procedure, single cells, as well as small clusters consisting of up to five cells, were obtained.

Throughout the preparation procedure, as well as during dye loading, we employed a physiological solution containing: 130 mM NaCl, 4.7 mM KCl, 1.3 mM CaCl_2_, 1 mM MgCl_2_, 1.2 mM KH_2_PO_4_, 10 mM glucose, 10 mM Hepes, 0.01% trypsin inhibitor (soybean) and 0.2% bovine serum albumin (pH = 7.4 adjusted with NaOH). During fluorescence determinations, trypsin inhibitor and bovine serum albumin were not included in the medium. In experiments where Ca^2+ ^free medium is indicated, Ca^2+ ^was omitted from the extracellular solution and 250 μM EGTA was added.

Cell viability was not significantly changed by the experimental treatments as assayed by trypan blue exclusion test and was greater than 95%. After loading with fluorescent dyes, cells were kept at 4°C until use and the experiments were performed within the next 4 hours.

### Determination of intracellular free Ca2+ concentration ([Ca^2+^]_i_)

Freshly isolated mouse pancreatic acinar cells were loaded with fura-2- acetoxymethyl ester (4 μM) at room temperature (23-25°C) for 40 min as previously established [1]. In single cell experiments, monitorization of Ca^2+^-dependent fluorescence signals was carried out by placing small aliquots of cell suspensions into a coverslip mounted on an experimental perfusion chamber, and placed on the stage of an epifluorescence inverted microscope (Nikon Diaphot T200, Melville, NY, USA). The cells were continuously superfused with Na-Hepes buffer. For fluorescence change determination, an image acquisition and analysis system for video microscopy was employed (Hamamatsu Photonics, Japan). Cells were alternatively excited with light from a xenon arc lamp passed through a high-speed monochromator (Polychrome IV, Photonics) at 340/380 nm. Fluorescence emission at 505 nm was detected using a cooled digital CCD camera (Hisca CCD C-6790, Hamamatsu) and recorded using dedicated software (Aquacosmos 2.5, Hamamatsu Photonics, Japan). All fluorescence measurements were made from areas considered individual cells. All stimuli were dissolved in the extracellular Na-Hepes buffer and applied directly to the cells in the perfusion chamber. Experiments were performed at room temperature (23-25°C).

In cell suspensions experiments, monitorization of changes in fluorescence signals was performed by placing 2 ml aliquots (10^6 ^cells/ml) of dye-loaded pancreatic acinar cells into a cuvette in a fluorescence spectrophotometer (Cary Eclipse, mod. EL 0210-6479 from Varian, U.S.A.). Cells were continuously stirred and experiments were performed at 37°C. All stimuli were dissolved in the extracellular Na-Hepes buffer, with or without Ca^2+^, and were directly added into the cuvette to yield the final concentration required.

For comparisons of the effects of CCK-8 on [Ca^2+^]_i_, several values of the Ca^2+ ^response were calculated: (1) the peak [Ca^2+^]_i _response, which was calculated at the maximal value achieved after stimulation of cells with the agonist; (2) a "steady-state level", calculated 5 minutes after application of the stimulus; (3) the total Ca^2+ ^mobilization after the stimulus was estimated as the integral of the rise in [Ca^2+^]_i _over the basal values for 8 minutes after addition of CCK-8; (4) and, finally, the rate of decay of [Ca^2+^]_i _to basal values, after application of the stimulus, which was calculated using the constant of the exponential decay. Traces were fitted to the equation Y = A × e^-KX ^+ plateau, where k is the constant of the exponential decay, X is the time and A is the span.

We can discard an effect of ethanol on fura-2- emission fluorescence that could induce artefacts in the determination of [Ca^2+^]_i_. In our experimental conditions, the concentration of ethanol employed did not influence fura-2-derived fluorescence. Furthermore, no apparent morphological changes of pancreatic cells were observed that could introduce errors in Ca^2+ ^measurements in our experimental conditions (data not shown).

Results for [Ca^2+^]_i _are expressed in nM ± SEM (n), where n is the number of independent experiments, following the calibration method proposed by Grynkiewicz et al. [39].

### Determination of ROS formation

Free radical production was measured by incubating freshly isolated mouse pancreatic acinar cells in the presence of CM-H2DCFDA (10 μM) for 40 min at room temperature (23-25°C). CM-H_2_DCFDA is a stable non-fluorescent molecule that passively diffuses into cells, where the acetate can be cleaved by intracellular esterases to produce a polar diol that is well retained within the cells. The diol can then be oxidized by ROS to a fluorescent form. This dye has been proved to be an excellent probe for determination of ROS production [20].

Red-ox state of cells was determined by exciting CM-H_2_DCFDA-loaded cells at 488 nm and detection of emitted fluorescence was performed at 530 nm. Cells were continuously stirred and maintained at 37°C. All agents tested were dissolved in the extracellular Na-Hepes buffer and directly added into the cuvette to yield the final concentration required. Data are expressed as absolute values of fluorescence previously normalized to 1 with respect to basal fluorescence.

### Statistical analysis

Statistical analysis of data was performed by one-way analysis of variance (ANOVA) followed by Tukey post hoc test, and only *P *values < 0.05 were considered statistically significant. For individual comparisons and statistics between individual treatments we employed Student's *t *test and only *P *values < 0.05 were considered statistically significant.

## Abbreviations

[Ca^2+^]_i_: cytosolic free Ca^2+ ^concentration; CCK-8: cholecystokinin octapeptide; CM-H_2_DCFDA: 5-(and-6)-chloromethyl-20,70-dichlorodihydrofluorescein diacetate acetyl ester; EGTA: ethylene glycol-bis(2-aminoethylether)-N,N,N'N'-tetraacetic acid; ER: endoplasmic reticulum; Fura-2/AM: fura-2 acetoxymethyl ester; IP_3_: inositol 1,4,5-trisphosphate; 4-MP: 4-methylpyrazole; ROS: reactive oxygen species.

## Authors' contributions

MFS carried out the experiments for [Ca^2+^]_i _determinations in single cell. ACV carried out the experiments for [Ca^2+^]_i _and ROS determinations in cells suspensions. AG conceived the study, its design and wrote the manuscript. GMS collaborated in the design of the study and helped to draft the manuscript. All authors read and approved the final manuscript.
